# Microdevices for examining immunological responses of single cells to HIV

**DOI:** 10.1042/BSR20140097

**Published:** 2014-08-18

**Authors:** Jonghoon Choi, Yoon Jeong, Hyung-Seop Han, Kwan Hyi Lee

**Affiliations:** *Department of BioNano Technology, Graduate School, Hanyang University, Seoul, Korea; †Department of BioNano Engineering, Hanyang University ERICA, Ansan, Korea; ‡Center for Biomaterials, Biomedical Research Institute, Korea Institute of Science and Technology, Seoul, Korea

**Keywords:** cellular immune response, HIV, integrative analysis, microfluidics, microwells, single-cell analysis, T cells, vaccines, ART, antiretroviral therapy, ELISpot, enzyme-linked immunosorbent spot, HLA, human leucocyte antigen, ICS, intracellular cytokine staining, IFN, interferon, IL, interleukin, LTNP, long-term non-progression, MCP, monocyte chemotactic protein, MHC, major histocompatibility complex, PBMC, peripheral blood mononuclear cell, SIV, simian immunodeficiency virus, TCR, T-cell receptor, T_H_1, T-helper 1 cell, TNF, tumour necrosis factor, T_reg_, regulatory T cell

## Abstract

More than 60 million people in the world have been diagnosed with HIV infections since the virus was recognized as the causative agent of AIDS in the 1980s. Even though more than half of the infected patients have died, effective disease treatment and prevention measures have not been established. ART (antiretroviral therapy) is the only proven HIV treatment that sustains the suppression of patient viraemia. Current routine approaches to treat HIV infections are targeted at developing vaccines that will induce humoral or cell memory immune responses. However, developing an effective vaccine has been challenging because the HIV mutates rapidly, which allows the virus to evade immune surveillances established against the previous strain. In addition, the virus is able to quickly establish a reservoir and treatment is difficult because of the general lack of knowledge about HIV immune response mechanisms. This review introduces common disease symptoms and the progression of HIV infection with a brief summary of the current treatment approaches. Different cellular immune responses against HIV are also discussed, with emphasis on a nanotechnology research that has focused on probing T-cell response to HIV infection. Furthermore, we discuss recent noteworthy nanotechnology updates on T-cell response screening that is focused on HIV infection. Finally, we review potential future treatment strategies based on the correlations between T-cell response and HIV infection.

## INTRODUCTION

HIV is the causative agent of AIDS. HIV infection is now regarded as a pandemic in humans [1]. Since its discovery in the early 1980s, AIDS has killed more than 30 million people worldwide [2]. HIV is known to infect a variety of immune response cells such as CD4**^+^**, CD8**^+^** T lymphocytes, macrophages, dendritic cells and natural killer cells. Extensive study of the infection mechanism has revealed that HIV enters the immune cells by HIV envelope glycoprotein conjugation, which recognizes the receptor molecules on the immune cells [3]. Once HIV enters the immune cell, the cell membrane interacts with the viral envelopes. Then, HIV releases its viral capsids inside the cell for viral replication [4]. ART (antiretroviral therapy) is the only currently proven therapy that can reduce the mortality associated with human HIV infection [5–8]. However, ART does not sufficiently remove the virus from the host and is accompanied by incomplete CD4^+^ T cell recovery. Clinical risk factors such as cardiovascular diseases [9] including myocardial infarction [10,11], have also been associated with ART. Most importantly, ART is too expensive for developing countries, where one-third of all AIDS-related human deaths have occurred.

This review addresses the therapeutic potential of HIV immunotherapy with a particular focus on the use of multifunctional T cells. In addition, this report also reviews high-throughput, integrated single-cell analysis techniques for the discovery of multifunctional T cells, which have shown promise in controlling HIV progression.

## HIV TRANSMISSION AND PROGRESSION

The progression of HIV is analysed by measuring the level of viraemia and the amount of T cells, and is categorized into four stages: virus incubation, transmission/migration, acute infection and the symptomatic stage. The initial virus incubation stage is difficult to assess before an individual is symptomatic since it usually occurs without clinical manifestations. Thus, studies on SIV (simian immunodeficiency virus) infection in macaques have provided an animal model that describes the initial transmission and disease propagation mechanisms of HIV-1 in humans [12,13]. The SIV model described CD4^+^ cells at the portal of entry (e.g. genital mucosal tissue) as the first targets of viral replication. The local expansion of SIV in both resting and activated CD4^+^ T cells takes place during the first week of infection. Then, the virus rapidly migrates to the gut-associated lymphoid tissue, where it induces massive depletion of memory CD4^+^ T cells, and establishes a self-propagating infection in secondary lymphoid organs. An acute infection stage is reached, approximately 2–4 weeks following the virus transmission, in which the infected individuals experience clinical manifestations such as cold-like symptoms including fever and sore throat induced by pharyngitis [14,15]. This stage is also characterized by malaise, a condition of an abnormally high proportion of monocytes in the blood and prevalent lymph node malfunctions. Physiologically, it induces not only massive viral replication in the absence of immune response, but also causes a 100-fold increase in HIV RNA counts [14,15]. Furthermore, it results in a significant loss of CD4^+^ T cells in peripheral blood that eventually leads to the symptomatic stage of infection (i.e. AIDS) [16]. After the acute infection stage, an asymptomatic stage reappears and can last for a lifetime unless the patient reaches the last infection stage or AIDS.

## CELL-MEDIATED HIV TREATMENT SHOULD REPLACE CONVENTIONAL THERAPEUTIC METHODS

Although ART has been successful in treating HIV infection [17,18], this treatment is expensive which greatly reduces the accessibility for patients that need it. Furthermore, in most developing countries, ART is initiated after the disease has progressed, and these individuals often experience one or more complications. Once ART is initiated, immunosuppressed individuals with infections or low CD4^+^ T-cell counts are at risk for immune reconstitution inflammatory syndrome [19]. Thus, improved therapies have been developed to minimize the side effects, enhance drug interaction with the virus, and increase drug resistance to viral mutations.

Cost-effective vaccinations have been proposed as an optimal solution to prevent HIV-1 infection in developing countries. Vaccine treatments were first designed with either live attenuated virus or whole-killed virus or subunits of virus. However, the US Food and Drug Administration have not approved the uses of these vaccine techniques for HIV vaccine development due to the safety concerns and the inability of the vaccine to induce widely applicable neutralizing antibody responses [20,21]. Today's approaches for vaccine development utilize plasmid DNA or live recombinant vectors engineered to express HIV-1 antigens [22,23]. Although these gene delivery approaches have appeared promising, they are not desirable in actual cases of HIV-1 infection because DNA vaccines usually require high doses to elicit clinically effective immune responses. Vaccine approaches that are currently available are focused on sustaining the disease at a non-symptomatic stage and include both humoral and cellular level treatments. For example, a phase IIb human trial (the Step Vaccine trial) was conducted to determine if T-cell vaccine reduces viraemia or the infection rate [24]. The efficacy of cell-mediated immune responses was assessed using adenovirus five-based vaccine (MRKAd5) that contained *gag*, *pol* and *nef* inserts. Although the MRKAd5 vaccine elicited more apparent T-cell response than the combinatory vaccination with ALVAC and AIDSVAX, this vaccine did not reduce the early plasma viral level or prevent HIV infection. Recently, the largest vaccine trial in human subjects (RV-144 Thai Trial) reduced the risk of HIV infection by administering ALVAC-HIV (recombinant canarypox vector vaccine) and AIDSVAX B/E (a recombinant glycoprotein 120 subunit vaccine) prime-boost regimen [25]. This study showed a limited but significant protection from HIV acquisition. A vaccine efficacy of 31.2% was demonstrated in 16395 subjects (*P* = 0.04), without substantial changes in the degree of viraemia or the CD4^+^ T-cell count. Although the reported efficacy is modest and insufficient against HIV acquisition, this combination of recombinant avian poxvirus live vector vaccine and subsequent booster injections of a glycoprotein 120 provides a potential HIV treatment for lower-risk participants. Prior to the RV-144 Thai trial, a phase 2 trial of this prime-boost platform in 2004 induced humoral and cellular immune response that lead to large-scale responses [26]. However, administration of the envelope glycoprotein subunit alone failed to show protective efficacy in phase 3 trials [27,28].

To date, most vaccine strategies typically generate only 100–1000 specific memory CD8^+^ T cells per million lymphocytes in the blood [29]. The rapid viral mutation of HIV is another major obstacle in vaccine development, particularly because the significant mutation of the *Env* gene amino acid sequence allows the virus to evade recognition by immune cells [30,31]. Furthermore, insufficient information of the immune defence mechanism against HIV-1 infection makes vaccine development an even more challenging task. Cell-mediated immunotherapeutic approaches have been introduced to overcome the limitations associated with current therapeutic modules.

## CELL-MEDIATED THERAPY POTENTIAL

Humoral immunotherapy (e.g. neutralizing antibodies) is one possible approach to treating HIV [32–35]. As previously discussed, HIV-1 Env glycoprotein protects the virus from recognition by immune antibodies. Although there are some broadly reactive antibodies that can compete with glycoprotein on HIV-1 Env's CD4-binding sites, the CD4-binding site is only partially accessible to the antibodies, which makes it difficult for humoral approaches [36,37].

**Figure 1:**
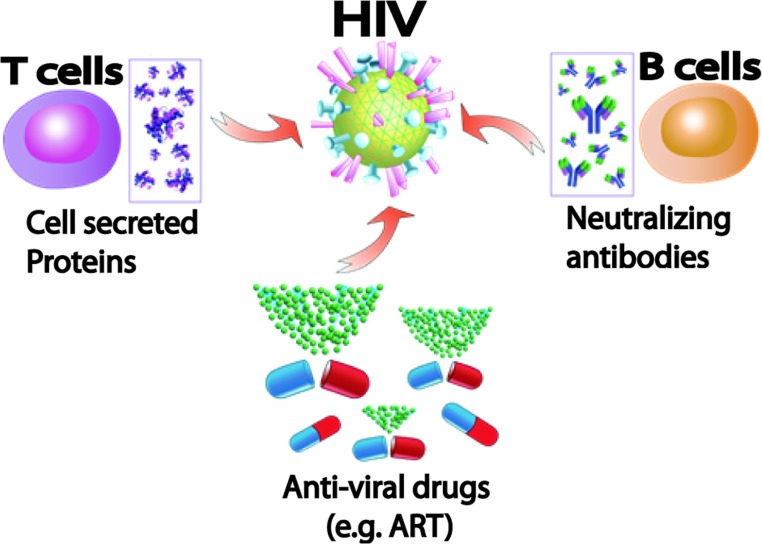
Cellular and drug-driven approaches in HIV treatment

The cellular level immune response is as important as the humoral level immune response in defence against viral infections (see Figure 1). T-cells promote proliferation of the other immune cells, enhance immune responses and deliver effector functions by cytokine secretion. Hence, virus-specific T-lymphocyte responses are critical in controlling HIV-1 progression at a cellular level. For example, HIV-specific CD8^+^ T cells have impaired cytolytic function. Moreover, the reports have indicated that the increased frequency of activated lymphocytes, manifested by up-regulation of activation marker CD38 on CD8^+^ T cells, is correlated with displaying HIV disease progression and viral replication [38,39]. Specifically, CD4^+^ T cells have a role in LTNP (*long-term* non-progression) to AIDS [40]. Vaccination in the absence of CD4^+^ T cells reduced CD8^+^ T-cell-mediated protection after SIV infection [41]. Therefore it is suggested that the T-cell contribution is important in HIV disease suppression. In addition to the limitations of conventional vaccine approaches and the challenges for neutralizing antibody production, inevitable participation of T cells in HIV retention has led researchers to consider a cell-mediated approach such as an adaptive transfer of immune cells.

For this reason, combining early viral suppression using ART with cellular immunotherapy is thought to augment HIV-specific immune response. For example, IL (interleukin)-2, IL-7 and IL-15 have been identified as the primary regulators of T-cell homoeostasis and may be considered as a immunotherapeutics to support vaccine-promoted T-cell responses for the treatment of HIV/SIV [42]. A phase I human trial demonstrated that a single administration of IL-7 affected maturation of circulating human B cell [43]. In addition, a phase I/IIa study demonstrated the immunological effect of recombinant human IL-7 in human subjects with insufficient immune restoration [44]. Repeated administration of T-cell regulator IL-7 (i.e. eight subcutaneous injections of two doses of IL-7, 3 and 10 μg/kg) showed that naïve and central memory CD4^+^ and CD 8^+^ T cells were significantly increased in a dose-dependent manner. Several parameters such as effective dose, dosing interval and adverse side effects are under investigation to develop more effective immunotherapy [42]. Therefore the combination therapy would strengthen T-cell responses by recovering both humoral and cellular immune responses and thus could replace the current life-long drug therapy method by enhancing viral control.

## IMMUNOLOGIC FUNCTIONS IN HIV-1 SUPPRESSION

Both CD4**^+^** and CD8**^+^** T-cell activities are crucial in immune responses to HIV-1. Research has been focused on how these T-cells react and function under HIV-1 infection [45,46]. T cells have heterogeneity and appear to have very diverse functions as a group. Functional subsets of CD4^+^ T cells, such as T_H_1 (T-helper 1) and T_H_2 (T-helper 2) cells, are defined by the cytokines they produce, such as IFN-γ (interferon-γ) and IL-4, respectively, or by interaction with other cells, including follicular helper T cells known as B-cell helper cells, pro-inflammatory T_H_17 (T-helper 17) cells and T_reg_ (regulatory T) cells which regulate autoimmune disease and the immune system by suppressing T-cell activities [47–49]. Each individual T cell is comprised a particular subset of T-cell functions. Proinflammatory cytokines [e.g. IL-2, TNF-α (tumour necrosis factor-α), IL-12 and -13], chemokines [e.g. CXCL10 (CXC motif chemokine ligand 10)] and lipopolysaccharide are thought to be involved in modulating HIV-specific immune response. For example, the frequency of IFN-γ-producing T cells has been adapted to assess vaccine-induced responses that are specific for HIV-1 infection [50]. TNF-α elicits the death of a variety of intracellular infectious viruses, including HIV-1 [51]. Also, IL-2 promotes the expansion of CD4**^+^** and CD8**^+^** T cells and works synergistically with other cytokines, thereby increasing the immune responses to HIV-1 [52]. In addition, IL-21, produced primarily by the CD4^+^ T cells, plays a crucial role in promoting the humoral response. IL-21 is believed to promote terminal differentiation of B cells, increase Ig production and to help develop T_H_17 [53]. A list of cytokines, chemokines and defensins involved in HIV suppression is thoroughly reviewed in the article by Alfano and Poli [54].

## T-CELL RESPONSE AND THE IMPORTANCE OF LONG-TERM NON-PROGRESSORS

Isolated virus from LTNPs exhibits normal replication kinetics and lacks any genetic insertions or deletions, implying that host factors are responsible for durable control of HIV [55]. Extremely low genetic diversity within the *env* gene of plasma virus from those isolated viruses suggests limited viral envelope replications and mutations. These findings may indicate that the key factors promoting long-term maintenance of the HIV-1 lie within the host-mediated control of competent virus replication [56].

It has been suggested that T-cell-mediated HIV control does not necessarily correlate with the frequency or the magnitude of the response. Despite the critical role of CD8^+^ T cells in cytolytic activities of HIV infection, the progression does not show a correlation with CD8^+^ T-cell counts, whereas HIV progression is highly related to a decline in CD4^+^ T-cell count. For this reason, many researchers have focused on the ‘quality’ of T-cell response, a combination of functions that T cells are able to carry out, instead of the frequency of T-cell populations or phenotype characterization [57]. Furthermore, owing to the complexity of a native immune system, the synergistic effect produced by interactions between T cells and other cells is also important. For example, CD4^+^, CD25^+^ and T_reg_ cells are involved in suppression of both CD4^+^ and CD8^+^ T cells [58]. Exposure of T_reg_ cells to HIV virus causes the T_reg_ cells to have a 2–5-fold increase in suppressive activities. Infected T_reg_ cells also become resistant to induced apoptosis like other resting CD4^+^ T cells.

**Figure 2:**
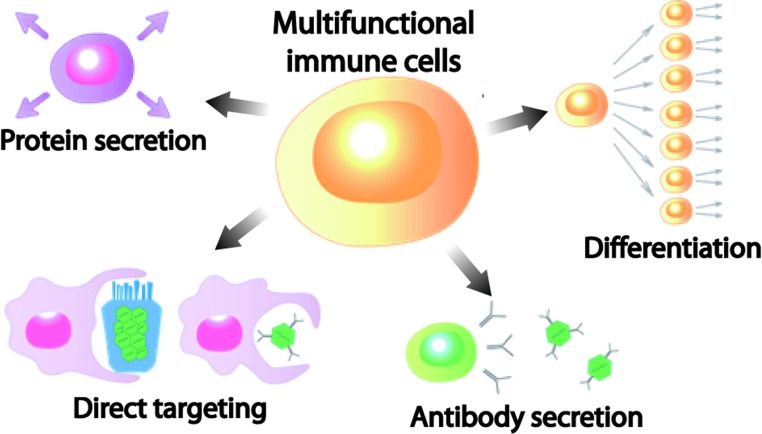
Possible pathways of reaction for multifunctional immune cells against HIV

In this respect, the cell behaviour in LTNPs is of interest in understanding the mechanism of HIV suppression by humoral and cellular immune responses. Within the group of LTNP individuals, individuals who maintain an undetectable plasma viraemia are called elite controllers. Comparing T-cell responses of the LTNPs and those of the progressors will provide information about immune response involved in HIV suppression. LTNPs are thought to have a better T-cell response than progressors, including better proliferative and cytolytic capacity, multifunctionality and genetic factors [e.g. possession of HLA (human leucocyte antigen) class I alleles that select for key epitopes]. Studies have shown that elite controllers have high frequencies of HIV-specific CD4^+^ and CD8^+^ T cells compared with highly active ART suppressed subjects or non-controllers [59]. Therefore in this review, we focused on the importance of T cell multifunctionality in immunosuppression of HIV (see Figure 2) and recently developed integrative nanotechnologies that are ongoing actions to integrate a close linking of nanotechniques, including chemical, biological and medical sciences for analysing single T-cell functions. Integrating measurements can be achieved using nanotechnologies, such as the microfluidic system. In this manner, this integrative approach might offer a new clue that could lead to better treatment of HIV infections.

## MULTIFUNCTIONAL T CELLS FINE-TUNING THE IMMUNE RESPONSE

T-cell characteristics have been proposed as a possible solution to HIV-1 infection because of the ability of T cells to counteract the low level of viraemia maintained by HIV-1 LTNPs [60]. There is a consensus that the improved control of HIV is associated with increased prevalence of multifunctional CD4^+^ T cells that produce two or more different cytokines simultaneously, although the mechanisms of the disease control are still unclear. It has been suggested that there is a distinct population of functional cytokines secreted from T cells and this cytokine population is a more potent effector. A study using multiparameter flow cytometry showed that the LTNPs have an increased prevalence of T cells that express IL-2 only or both IL-2 and IFN-γ, whereas the progressors had CD4 T^+^ cells that expressed only IFN-γ [61]. Several factors account for the optimal protective function of multifunctional CD4^+^ or CD8^+^ T cells. Multifunctional T cells secrete more IFN-γ on a per-cell basis (~10-fold greater), and the cells demonstrate more efficient cytolytic activity when both IFN-γ and TNF-α are released from the same T cell [61,62]. Observed CD8^+^ T cells that secreted both INF-γ and TNF showed more enhanced levels of cytolytic activity [63]. Elite controllers were also able to produce a stronger and wider breadth of cytokines and chemokines, including antiviral factors after HIV stimulation; PBMC (peripheral blood mononuclear cell) from elite controllers produced significantly more IFN-γ, granulocyte -macrophage colony-stimulating factor (GM-CSF), interferon-induced protein-10 (IP-10), MCP-10 (monocyte chemotactic protein-10), MCP-3, TNF-α and IL-2 than the HIV seronegative individuals after p55 stimulation. In comparison, PBMCs obtained from progressors produced the lowest levels of HIV-induced cytokines.

There are various factors that generate multifunctional T cells. First, the frequency of T cells, antigen sensitivity of TCRs (T-cell receptors) and the amount of antigen load required for TCR activation are effectors of the multifunctional T-cell response. Studies have shown that HIV-specific CD8^+^ T cells with high antigen sensitivity display multifunctional secretory profiles [64]. Experiments with the T_H_1 CD4^+^ cell model in mice indicated that multifunctionality appeared with an increased concentration of the ligand [65]. In the cytomegalovirus-specific CD4^+^ memory human T-cell model, an increasing co-stimulation increased the frequency of responding CD4^+^ T cells, and IFN-γ producing cells also produce IL-2 with increased co-stimulation. However, it is important to note that heterogeneity in the co-stimulation threshold amount existed within a sample. Secondly, a lineage is an important determinant of multifunctionality. The lineage where the effector T cell has been derived from could be also an important factor; CD8^+^ T cells engineered from naïve rather than memory subsets had better efficacy in adoptive immunotherapy [66]. Upon antigen stimulation, naïve CD4^+^ T cells become effector cells such as T_H_1 or T_H_2 cells. When antigens are cleared, a smaller population of cells remains as central memory cells and reside in lymphoid organs, or effector memory cells. These cells reside in peripheral tissues to respond to similar future antigenic attacks. Therefore precise modulation in these determining factors could be a promising strategy to augment T-cell multifunctionality.

## KEY STRATEGIES TO DEVELOP CELL-MEDIATED HIV TREATMENT

Despite recent developments, T-cell-mediated immunity is faced with various challenges associated with viral mechanisms. The virus tends to limit the T-lymphocyte responses by inducing mutations in T lymphocyte epitopes to escape from a cellular immune defence system [67]. Attempts to control viral replication by implementing diverse epitope-specific T-cell responses have failed due to immunodominance constraints [68]. In addition, the CD8^+^ T-lymphocyte responses are supposed to be deposited on a limited number of epitopes. Therefore while T-lymphocyte multifunctional immune responses are expected to be effective against HIV-1, the limitation of vaccine-elicited cellular immune responses makes it difficult to provide practical protection against the acquisition of HIV-1 infection. The successful isolation and improvement of a specific subset of T cells is the key to developing cell-mediated HIV treatment. The following discussion is focused on a functional screening and quantification of T-cell subsets that are effective in disease control.

## TECHNIQUES FOR LIMITING CONVENTIONAL MULTIPLEXED CHARACTERIZATION

ELISpot (enzyme-linked immunosorbent spot) and ICS (intracellular cytokine staining) are common immunoassays for quantification of HIV-specific immune cell response. ELISpot is a popular assay that detects low-frequency T-cell responses [69]. ELISpots were originally developed to quantitate memory B-cell responses secreting antibodies, and have subsequently been adapted for various tasks that allow the simultaneous detection of multiple antibodies. Especially, the identification and enumeration of cytokine-producing cells at the single-cell level HIV-specific immune response. However, this method requires a large number of cells and requires sacrifice of the cells for the assay. It does not retain the cell viability, and also requires a cost-intensive HLA types/epitopes typing procedure for antigen stimulation. A microfabricated platform using aptamer-modified electrodes is also available for simultaneous cytokine detection. Cytokine capture using micropatterned, aptamer-modifed electrodes allows for multiplexed detection of TNF-α and IFN-γ secreted from T cells fed and bound by microfluidic channels. In this platform, cytokine secretion dynamics (e.g. quantity and rate) can be measured [70]. However, as in the conventional ELISpot method, neither of these assays is suitable for cell-type specific (e.g. central memory or effector) protein detection.

The ICS method allows for rapid enumeration of cytokine-producing T cells in a large pool of cells [71]. A great advantage of flow cytometry is the possibility of simultaneous phenotype characterization of antigen-specific T cell by multicolour flow cytometry. The multiparameter flow cytometry is able to characterize multiple functions of individual T cells [61]. ICS can be used in combination with other flow cytometry protocols–the so-called secretion assay–for immunophenotyping using cell surface markers or with MHC (major histocompatibility complex) multimers to detect an antigen-specific response, making it an extremely flexible and versatile method. Recent studies using multiparameter flow cytometry have shown different functional diversity of T lymphocytes in terms of cytokine secretion, degranulation, proliferation and effector functions [62]. For example, combined fluorescent peptide and MHC tetramers have increased the number of simultaneous T-cell sorting specialties by up to 15. Despite the multiplexity of the flow cytometry-based method, ICS has some intrinsic drawbacks. First of all, ICS does not retain cell viability, and thus, recovery of cells for clonal expansion is difficult. In addition, since a protein transport inhibitor is added to retain the cytokines within the cell, it is difficult to determine whether the detected cytokines are intracellular, secreted or membrane-bound [72]. Moreover, dynamic monitoring of an individual cell is difficult since the secretions of the cells upstream may affect others. Lastly, since a single pathogenic protein can give rise to several epitopes that bind different MHC molecules that are characteristic of each individual, there is a need for a high-throughput screening system that incorporates cell cultures and antigen presentation via professional antigen-presenting cells. To overcome such obstacles for the effective screening of HIV-specific immune responses, methods to monitor multiple immune response markers consistently at single-cell resolution–as demonstrated in integrated single-cell analytic applications–have been developed.

## INTEGRATIVE SINGLE-CELL ASSAY

**Figure 3:**
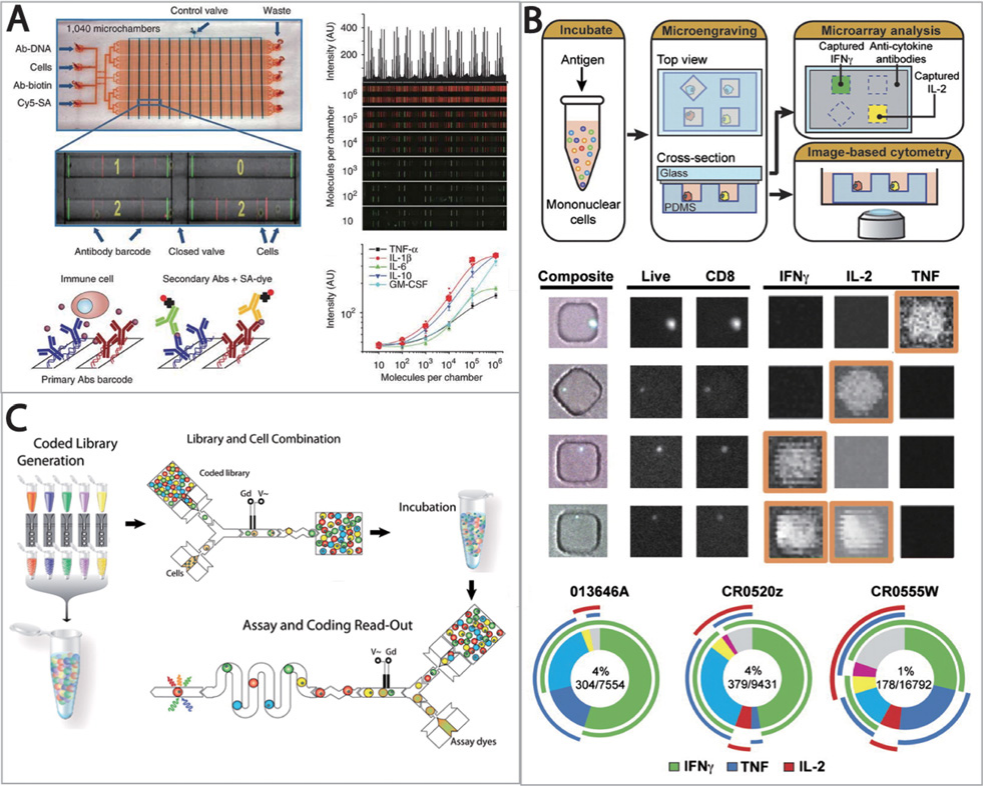
Integrative single-cell assays for multiplexed analysis of individual immune cell function (**A**) Microfluidic assay based on a DNA-encoded antibody library. Reprinted by permission from Macmillan Publishers Ltd: Nature Medicine (Ma, C., Fan, R., Ahmad, H., Shi, Q., Comin-Anduix, B., Chodon, T., Koya, R.C., Liu, C.C., Kwong, G.A., Radu, C.G. et al. A clinical microchip for evaluation of single cells reveals high functional heterogeneity in phenotypically similar T cells. **17**, 738–743), copyright (2011). (**B**) Protein-printed nano-microwell assay fabricated by the microengraving technique. Reprinted by permission from National Academy of Sciences, USA: Varadarajan, N., Kwon, D.S., Law, K.M., Ogunniyi, A.O., Anahtar, M.N., Richter, J.M., Walker, B.D., Love, J.C. Rapid, efficient functional characterization and recovery of HIV-specific human CD8^+^ T cells using microengraving. Proc. Natl. Acad. Sci. U.S.A. **109**, 3885–3890. Copyright (2012) National Academy of Sciences, USA. (**C**) Nano-micro droplet assay using a coded droplet library. Reprinted by permission from National Academy of Sciences, USA: Brouzes, E., Medkova, M., Savenelli, N., Marran, D., Twardowski, M., Hutchison, J.B., Rothberg, J.M., Link, D.R., Perrimon, N. and Samuels, M.L. Droplet microfluidic technology for single-cell high-throughput screening. Proc. Natl. Acad. Sci. U.S.A. **106**, 14195–14200. Copyright (2009) National Academy of Sciences, USA.

Immune cells found in the clinical samples are diverse in terms of their functions, clonotypic breadth and lineage [73]. Antigen-specific cells diversify by effector function, differentiation stage and migratory capacity, which may differ even within a single clone [74,75]. However, conventional techniques do not account for this inherent heterogeneity; they only capture a mean response from a heterogeneous population of cells, and thus, do not provide the in-depth analysis of disease mechanisms, proteomic interactions and the clonotypes of the immune response. Therefore more comprehensive and multiplexed analysis at single-cell resolution is important. Some examples of reported integrative single-cell assays are discussed here. Three major categories of integrative, novel single-cell assays are microfluidic assays, nano-microwell assays and nano-micro droplet assays (see Figure 3) [76–78].

Ma et al. [76] reported a microfluidic platform designed for highly multiplexed and quantitative measurements of secreted proteins from a single cell (Figure 3A). This microchannel-based system is reliable for assessing high functional heterogeneity at the single-cell level. Once a small number of cell populations are introduced into the system, cells are isolated in each microchamber (0–40 cells per chamber). The system utilizes a DNA-encoded antibody library barcode array to capture the secreted proteins from single cells. Each barcode-encoded glass substrate is coated with a distinctive antibody. Fluorescence intensities for each protein from each microchamber are analysed for quantification. This single-cell barcode chip system showed a multiplexing capacity, by quantifying 12 different inflammatory cytokines and determining heterogeneity in active tumour antigen-specific cytotoxic T lymphocytes. In addition, this system only requires a small (~1×10^4^ cells) number of cells to detect less than a thousand copies of proteins. Therefore this platform is advantageous because of its smaller sample size, higher sensitivity and superior multiplexing capacity that could be applied to analyse the cellular level immune responses of HIV-specific cells.

The second approach would be a soft lithographic technique that utilizes microengraving that has also been used to produce a set of replicated protein-printed microarrays (Figure 3B) [77]. Each spot on the microarray is composed of the proteins secreted by a single cell. General fabrication steps are as follows. Once the polydimethylsiloxane microwell array is fabricated a series of photolithographic techniques is applied and a cell suspension is applied onto the microwells (0.1–1 nl each). The individual cell is deposited in each well and the array is placed onto a capture antibody-coated supportive glass slide. Secreted products from a cell are deposited on the surface of the glass slide. The identified individual cells can be recovered by micromanipulation for further *in vitro* characterization. Therefore this high-throughput system can perform identification, recovery and clonal expansion of cells that produce antigen-specific antibodies. The multiparametric datasets describe the antigenic specificity, isotype and affinity of the antibodies secreted from large numbers of individual primary B cells [79]. In addition, studies also reported characterization and recovery of HIV antigen-specific human CD8^+^ T cells from human subjects [72], as well as monitoring dynamic cytokine secretion from individual human CD3^+^ T cells from peripheral blood [73]

The third approach is to utilize a droplet-based microfluidic system that allows the single cells to be encapsulated in individual aqueous droplet with a volume of 1 pl–10 nl (Figure 3C) [78]. A sequential process of encapsulation, incubation, manipulation and analysis can be performed on the microfluidic device [78,80,81]. In these systems, the specific sequential steps of droplet screening are: (1) preparation of a coded droplet library by the combination of different concentrations of mitomycin C and fluorescent optical labelling; (2) mixing a library droplet and a cell-containing droplet; (3) on-chip incubation; and (4) measurement of each droplet's fluorescence for both assay and drug coding read-out. Therefore successful development of libraries that are associated with HIV antigen-specific cells may provide a feasible high-throughput platform for screening T-cell response.

To determine molecular level identification and gene signatures that encompass antigen specificity, a Fluidigm Biomark dynamic array could be introduced [82]. This qPCR-base single-cell gene-profiling technique can assay up to 96 genes from 96 individual cells in a single experiment. In the context of CD4^+^ T cells, transcription factor gene profiling correlated with cellular function may enable identification of the functional subsets of the antigen-specific T cells. In addition to these technical developments, various up-to-date clinical aspects of HIV immunotherapy, such as cytokine therapy, immune-modulating drugs and monoclonal antibodies, have also been actively studied and some of the recent advances are summarized in a review article by Kim and Han [83]. Recent developments in HIV nanomedicine are also addressed in the review articles [84,85]. Clinical aspects of nano/microfluidic technologies for diagnosing HIV are also addressed in a review article by Lee et al. [86].

In summary, we discussed the development of multiplexed formats of immunoassay techniques to study HIV-specific immune response with a particular focus on the microfabricated platforms for single-cell analysis. The integrative single-cell assay can be further developed as a promising and powerful cell-mediated immunotherapeutic platform to identify, characterize and sort a variety of T-cell functions and subsequently profile-specific T-cell responses to HIV.
